# Joint effect of visit-to-visit variability in LDL-cholesterol, HDL-cholesterol and HbA1c on cardiovascular and total mortality in patients with diabetes

**DOI:** 10.1186/s13098-022-00905-x

**Published:** 2022-09-19

**Authors:** Panpan He, Xiaoqin Gan, Qimeng Wu, Ziliang Ye, Sisi Yang, Yanjun Zhang, Huan Li, Chun Zhou, Yuanyuan Zhang, Mengyi Liu, Xianhui Qin

**Affiliations:** Division of Nephrology, National Clinical Research Center for Kidney Disease, State Key Laboratory of Organ Failure Research, Guangdong Provincial Key Laboratory of Renal Failure Research, Guangdong Provincial Clinical Research Center for Kidney Disease, Nanfang Hospital, Southern Medical University, Guangdong Provincial Institute of Nephrology, Guangzhou Regenerative Medicine and Health Guangdong Laboratory, Guangzhou, 510515 China

**Keywords:** Visit-to-visit LDL-C variability, Visit-to-visit HbA1c variability, Visit-to-visit HDL-C variability, Type 2 diabetes, Mortality

## Abstract

**Background:**

We aimed to investigate the joint effect of visit-to-visit variability (VVV) in low-density lipoprotein cholesterol (LDL-C), high-density lipoprotein cholesterol (HDL-C), triglycerides and glycosylated hemoglobin (HbA1c) on cardiovascular mortality and total mortality in patients with diabetes.

**Methods:**

Among 5194 participants with type 2 diabetes enrolled in the ACCORD lipid trial, VVVs of LDL-C, triglycerides, HDL-C, and HbA1c were assessed from baseline to 2 years of follow-up and expressed as coefficient of variation (CV). The study outcomes included cardiovascular mortality and all-cause mortality.

**Results:**

Over a median follow-up of 3.0 years from the end of variability measurements at years 2, there were 305 (5.9%) cases of all-cause mortality, of which, 144 were cardiovascular causes. The positive relations between LDL-C CV and cardiovascular mortality were significantly stronger among participants with higher HDL-C CV (*P* for interaction = 0.023), and higher HbA1c CV (*P* for interaction = 0.015). However, there were no significant interactions between LDL-C CV and triglycerides CV (*P* for interaction = 0.591). Similar trends were found for all-cause mortality. Consistently, there were graded trends in the risk of mortality with the increasing numbers of higher CV of the three variables: LDL-C, HbA1c, and HDL-C (*P* for trend = 0.008 for cardiovascular mortality, and *P* for trend < 0.001 for all-cause mortality).

**Conclusion:**

VVVs in LDL-C, HDL-C, and HbA1c may jointly affect the risks of cardiovascular and all-cause mortality in diabetes patients. Those with higher CVs of all three variables had the highest risks of cardiovascular and all-cause mortality.

**Supplementary Information:**

The online version contains supplementary material available at 10.1186/s13098-022-00905-x.

## Introduction

Diabetes has become a major health concern worldwide due to its high prevalence and the related huge burden of disability and mortality [1]. In 2019, International Diabetes Federation (IDF) estimated that about 463 million adults were suffering from diabetes worldwide [2]. As such, it is of great clinical and public health importance to identify more modifiable related factors to reduce the adverse outcomes in type 2 diabetes patients.

Recently, emerging evidence suggested that the long-term variability of many risk factors, including blood pressure [3, 5], lipids [6, 7], and glycosylated hemoglobin (HbA1c) [8, 9], were associated with adverse cardiovascular outcomes and mortality. These effects remained significant after adjusting for the mean levels of the parameters, suggesting that not only managing the absolute value but also reducing the fluctuation should be targeted to improve health outcomes. However, although it has been reported that the clustering of metabolic risk factors, such as dyslipidemia and glucose intolerance, may have a multiplicative effect on cardiovascular risks and death [10, 11], to date, few studies have systematically assessed whether there is a combined effect of low-density lipoprotein cholesterol (LDL-C) variability with the variabilities in triglycerides (TG), HbA1c and high-density lipoprotein cholesterol (HDL-C) on mortality risk.

To fill these aforementioned gaps in knowledge, in the current study, we used data from the Action to Control Cardiovascular Risk in Diabetes (ACCORD) trial to investigate the joint effect of visit-to-visit variabilities (VVVs) in LDL-C, TG, HDL-C, and HbA1c on the risk of cardiovascular and total mortality in patients with diabetes.

## Materials and methods

### Study design and population

The ACCORD trial design, inclusion criteria, subject characteristics, and main results have been previously described [12–15] (online study protocols: https://biolincc.nhlbi.nih.gov/studies/accord/). In brief, the ACCORD trial was a multicenter randomized trial that tested intensive blood glucose control compared with standard therapy in 10,251 patients with type 2 diabetes. Participants of the trial were 40 to 79 years of age with established cardiovascular disease or 55 to 79 years of age with evidence of atherosclerosis, left ventricular hypertrophy, albuminuria, or at least two of the following cardiovascular risk factors: hypertension, dyslipidemia, obesity, or current smoker. A subgroup of patients in the ACCORD study was enrolled in the ACCORD lipid trial and underwent randomization, in a 2-by-2 factorial design, to receive simvastatin plus either fenofibrate or placebo [15]. Randomization occurred between January 11, 2001, and October 29, 2005. End-of-study visits were scheduled between March and June 2009.

This report represents a post hoc analysis of data available for the ACCORD lipid trial. Of the 5518 participants of the ACCORD lipid trial, we excluded 202 participants who had less than 3 recorded lipids values in the first 2 years. Moreover, 122 participants dead within 2 years from enrollment were also excluded from the present analysis. Finally, a total of 5194 participants were included in the current study (Additional file 1: Fig. S1).

The ACCORD study was sponsored by the National Heart, Lung, and Blood Institute (NHLBI), and the protocol was approved by a review panel at the NHLBI, as well as by the institutional review board or ethics committee at each center. This post hoc analysis was approved by the Ethics Committee of Nanfang Hospital, Guangzhou, China.

### Measures of variability

A fasting plasma lipid profile was measured at the ACCORD central laboratory at 4, 8, and 12 months after randomization, annually thereafter, and at the end of the study. HbA1c was measured every 4 months.

Long-term VVVs of lipids were evaluated using three or more lipid measures from baseline to the first 2 years of follow-up, calculating individual participant coefficient of variation (CV) [16], average real variability (ARV), and variability independent of the mean (VIM). CV was calculated as the SD divided by the mean, and ARV as the average of the absolute differences between consecutive measurements [9, 17]. VIM was calculated as 100 * SD/Mean ^beta^, where beta is the regression coefficient, on the basis of the natural logarithm of SD on the natural logarithm of mean. In addition, this uncorrected VIM was corrected by the use of the formula [VIM uncorrected * (mean of CV)]/ (mean of VIM uncorrected) [4, 6]. The CV was used as the primary variability measure. Especially, the mean and variability of HbA1c were calculated using the data from the 4th month to the first 2 years of follow-up.

### Outcomes

The study outcomes were all-cause and cardiovascular mortality. Cardiovascular mortality included deaths from myocardial infarction, heart failure, arrhythmia, invasive cardiovascular interventions, cardiovascular causes after non-cardiovascular surgery, stroke, unexpected death presumed to be from ischemic cardiovascular diseases occurring within 24 h after the onset of symptoms, and death from other vascular diseases.

### Statistical analysis

Baseline characteristics are presented as means (standard deviation, SD) for continuous variables and proportions for categorical variables according to lipid treatment group (fenofibrate or placebo). The differences in population characteristics were compared using chi-square tests, or t-tests accordingly.

Cox proportional hazards models were used to examine the relationship of LDL-C VVV with the study outcomes, without and with adjustments for sex, glycemia treatment group (intensive or standard), lipids treatment group (fenofibrate or placebo), race, age, education, body mass index (BMI), diabetes duration, systolic blood pressure (SBP), smoking and drinking status, estimated glomerular filtration rate (eGFR) at baseline, as well as mean of HbA1c, HDL-C, TG, LDL-C during the first 2 years of follow-up. Possible modifications of the association between LDL-C CV (Q1-3 versus Q4) and CVD mortality or all-cause mortality were assessed for the HbA1c CV, HDL-C CV and TG CV during the first 2 years of follow-up.

In addition, the joint effect of LDL-C CV with other CVs, which significantly modify the association between LDL-C CV and mortality, on the risk of mortality was further examined.

A two-tailed *P* < 0.05 was considered to be statistically significant in all analyses. R software (version 3.5.0, http://www.R-project.org) was used for all statistical analyses.

## Results

### Characteristics of study participants

The flowchart of participants was presented in Additional file 1: Fig. S1. A total of 5194 participants were included in the final analysis, 4673(84.7%) of which had 5 times of LDL-C measurements in the first 2 years. The mean age of all participants was 62.7 years, and 69.3% were male. The mean LDL-C CV, HDL-C CV, TG CV, and HbA1c CV were 20.1 (SD: 10.2) %, 10.7 (SD: 7.0) %, 29.0 (SD: 15.0) %, and 6.7 (SD: 4.5) %, respectively.

The key baseline characteristics were similar in the 2 lipids treatment groups. However, participants in the fenofibrate group tend to have higher HDL-C CV, TG CV, HbA1c CV, higher means of HDL-C and HbA1c, and lower means of LDL-C and TG, during the first 2 years follow-up (Table 1).

**Table 1:** Characteristics of the patients at baseline and during follow-up by lipids treatment group

Characteristics	Total	Treatment		*P* value
		Fenofibrate	Placebo	
N	5194	2615	2579	
At baseline				
Age, year	62.7 (6.6)	62.7 (6.5)	62.8 (6.6)	0.827
Male, No. (%)	3602 (69.3)	1815 (69.4)	1787 (69.3)	0.927
BMI, kg/m^2	32.3 (5.3)	32.2 (5.3)	32.4 (5.4)	0.352
SBP, mmHg	133.8 (17.7)	133.8 (17.5)	133.9 (17.9)	0.850
DBP, mmHg	73.9 (10.7)	73.8 (10.6)	74.0 (10.9)	0.546
Current smoking, No. (%)	741 (14.3)	382 (14.6)	359 (13.9)	0.478
Current drinking, No. (%)	1279 (24.6)	649 (24.8)	630 (24.4)	0.732
Diabetes duration, year	10.6 (7.4)	10.6 (7.3)	10.7 (7.5)	0.767
Race/ethnicity, No. (%)				0.289
White	3417 (65.8)	1729 (66.1)	1688 (65.5)	
Hispanic	380 (7.3)	200 (7.6)	180 (7.0)	
Black	758 (14.6)	359 (13.7)	399 (15.5)	
Other	639 (12.3)	327 (12.5)	312 (12.1)	
Intensive glycemia, No. (%)	2584 (49.7)	1294 (49.5)	1290 (50.0)	0.699
Laboratory results				
HDL-C, mg/dL	38.1 (7.7)	38.0 (7.8)	38.2 (7.7)	0.278
LDL-C, mg/dL	100.5 (30.7)	99.9 (30.4)	101.1 (31.1)	0.156
Total cholesterol, mg/dL	175.2 (37.4)	174.8 (36.8)	175.7 (37.9)	0.402
Triglycerides, mg/dL	188.2 (112.9)	190 (111.4)	186.3 (114.4)	0.244
HbA1c, %	8.3 (1.0)	8.3 (1.0)	8.3 (1.0)	0.468
eGFR, mL/min/1.73 m^2	90.6 (25.7)	90.6 (24.7)	90.5 (26.6)	0.918
During the first two years				
LDL-C MEAN, mg/dL	92.0 (20.8)	91.4 (20.7)	92.6 (20.9)	0.034
HDL-C MEAN, mg/dL	39.7 (8.2)	40.1 (8.7)	39.3 (7.7)	< 0.001
HDL-C CV, %	10.7 (7.0)	12.1 (8.5)	9.4 (4.7)	< 0.001
TG MEAN, mg/dL	169.9 (96.8)	156.2 (89.2)	183.7 (102.2)	< 0.001
TG CV, %	29.0 (15.0)	30.7 (15.8)	27.3 (13.8)	< 0.001
HbA1c MEAN, %	7.1 (0.9)	7.2 (0.9)	7.1 (0.9)	0.035
HbA1c CV, %	6.7 (4.5)	7.0 (4.6)	6.4 (4.3)	< 0.001

Variables are presented as Mean (SD) or n (%)

*BMI* Body mass index, *SBP* systolic blood pressure, *DBP* diastolic blood pressure, *HDL-C* high-density lipoprotein cholesterol, *LDL-C* low-density lipoprotein cholesterol, *FPG* fasting plasma glucose, *eGFR* estimated glomerular filtration rate, *CV* coefficient of variation

### Interactions of LDL-C VVV and VVVs in TG, HbA1c or HDL-C on cardiovascular mortality and total mortality

Over a median follow-up of 3.0 years from the end of variability measurements at year 2, there were 305 (5.9%) cases of all-cause mortality, of which, 144 were cardiovascular deaths.

When LDL-C CV was assessed as quartiles, compared with those in the 1–3 quartiles (< 26.0%), significantly higher risks of cardiovascular mortality (HR, 1.78, 95% CI: 1.24–2.56) and all-cause mortality (HR, 1.61, 95% CI: 1.26–2.07) were found in those in the fourth quartile (≥ 26.0%) (Additional file 1: Table S1). Similar patterns were observed for alternative measures of LDL-C VVV, including ARV and VIM (Additional file 1: Fig. S2).

**Fig. 1:**
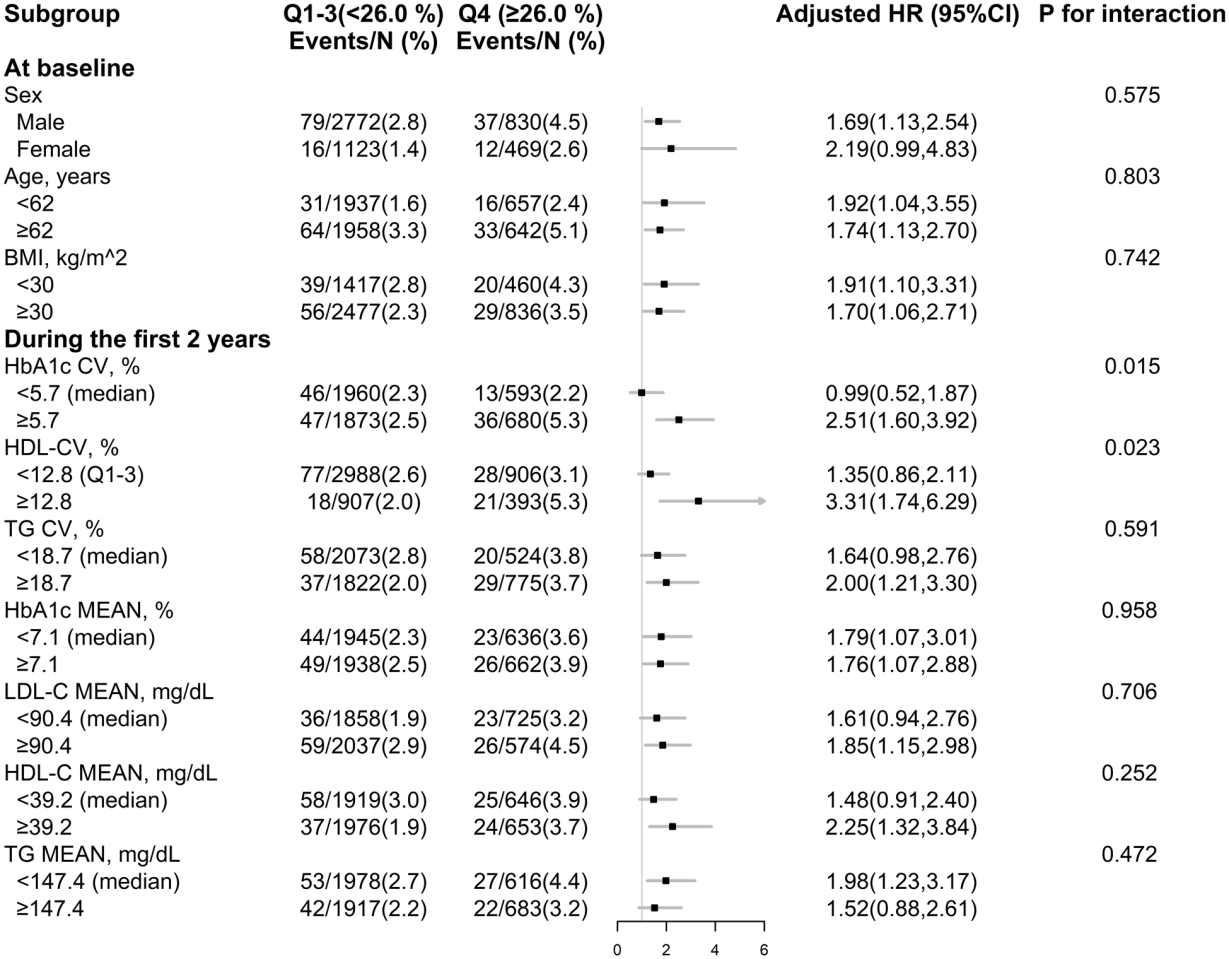
Stratified analyses by potential effect modifiers for the association between LDL-C CV and CVD mortality in various subgroups*. *Adjusted, if not stratified, for sex, glycemia treatment group, lipids treatment group, race, age, education, BMI, diabetes duration, systolic blood pressure (SBP), smoking and drinking status, estimated glomerular filtration rate (eGFR) at baseline, as well as mean of HbA1c, high-density lipoprotein cholesterol (HDL-C), triglyceride (TG), low-density lipoprotein cholesterol (LDL-C) during the first 2 years of follow-up

Stratified analyses were performed to assess the relation of LDL-C CV (Q1-3 *versus* Q4) and CVD mortality in various subgroups (Fig. 1, Additional file 1: Fig. S3). We first made the stratified analyses by quartiles of the variabilities and means in HbA1c, HDL-C and TG, and then combined the categories with relatively similar risks into one new group. Accordingly, for HDL-C CV, due to the stronger positive association in the fourth quartile, we combined the 1–3 quartiles into a new group. For HbA1c CV, due to the stronger and significant positive relations in the third quartile and fourth quartile, we chose the median as the cutoff point. Moreover, since TG CV and the means of HbA1c, HDL-C and TG did not obviously modify the association, the medians were chosen as the cutoff points (Fig. 1, Additional file 1: Fig. S3.

Accordingly, the positive relations of LDL-C CV and CVD mortality were significantly stronger among participants with higher HDL-C CV (≥ 12.8% [quartile 3]: HR, 3.31; 95% CI: 1.74–6.29; *versus* < 12.8% [quartile 3]: HR, 1.35; 95% CI: 0.86–2.11; *P* for interaction = 0.023), and higher HbA1c CV (≥ 5.7% [median]: HR, 2.51; 95% CI: 1.60–3.92; *versus* < 5.7% [median]: HR, 0.99; 95% CI: 0.52–1.87; *P* for interaction = 0.015) (Fig. 1). However, there was no significant interactions between LDL-C CV and TG CV (≥ 18.7% [median]: HR, 1.64 95% CI: 0.98–2.76; *versus* < 18.7% [median]: HR, 2.00; 95% CI: 1.21–3.30; *P* for interaction = 0.591). Similar trends were found in the relation of LDL-C CV with all-cause mortality (HbA1c CV: *P* for interaction = 0.044; HDL-C CV: *P* for interaction = 0.063; TG CV: *P* for interaction = 0.875) (Additional file 1: Fig. S3). Similar results were found in the crude and age, sex-adjusted models (Additional file 1: Table S2). Furthermore, none of the other variables, including sex, age, and BMI, significantly modified the relation of LDL-C CV with CVD mortality or all-cause mortality (Additional file 1: Fig.S3, Fig. S4).

### Joint effects of VVVs in LDL-C, HDL-C and HbA1c on cardiovascular and total mortality

Overall, there were graded trends in the risk of mortality with the increasing numbers of higher CV of the three variables. That is, compared with those with no higher CV of the three variables (LDL-C, HbA1c, and HDL-C), the HRs of CVD mortality and all-cause mortality were 0.84, 1.28, 2.73 and 1.00, 1.46, 2.69, respectively, for participants with one, two, and three higher CVs (*P* for trend = 0.008 for CVD mortality, and *P* for trend < 0.001 for all-cause mortality) (Table 2). Similar results were found in the crude and age, sex-adjusted models (Table 2).

**Table 2:** Associations between numbers of higher CV of the three variables (LDL-C, HbA1c, and HDL-C) and the study outcomes*

Number of higher CV	N	Event (%)	Crude models		Adjusted model 1		Adjusted model 2	
			HR (95%CI)	P value	HR (95%CI)	P value	HR (95%CI)	P value
CVD mortality								
0	1573	40 (2.5)	Ref.		Ref.		Ref.	
1	2195	49 (2.2)	0.86 (0.57,1.31)	0.483	0.90 (0.59,1.36)	0.614	0.84 (0.55,1.29)	0.429
2	1107	37 (3.3)	1.30 (0.83,2.03)	0.253	1.44 (0.92,2.26)	0.111	1.28 (0.81,2.03)	0.296
3	231	16 (6.9)	2.87 (1.61,5.13)	< 0.001	3.14 (1.75,5.61)	< 0.001	2.73 (1.48,5.05)	0.001
P for trend			0.003		0.001		0.008	
All-cause mortality								
0	1573	77 (4.9)	Ref.		Ref.		Ref.	
1	2195	112 (5.1)	1.02 (0.76,1.36)	0.900	1.06 (0.79,1.41)	0.705	1.00(0.74,1.34)	0.991
2	1107	81 (7.3)	1.47 (1.08,2.01)	0.016	1.61 (1.17,2.20)	0.003	1.46(1.06,2.01)	0.021
3	231	30 (13.0)	2.81 (1.84,4.28)	< 0.001	2.99 (1.96,4.56)	< 0.001	2.69(1.74,4.18)	< 0.001
P for trend			< 0.001		< 0.001		< 0.001	

^*^Model 1 adjusted for sex and age;

Model 2 adjusted for sex, glycemia treatment group, lipids treatment group, race, age, education, BMI, diabetes duration, systolic blood pressure (SBP), smoking and drinking status, estimated glomerular filtration rate (eGFR) at baseline, as well as mean of HbA1c, high-density lipoprotein cholesterol (HDL-C), triglyceride (TG), low-density lipoprotein cholesterol (LDL-C) during the first 2 years of follow-up

Similar results were also observed for alternative measures of LDL-C VVV, including ARV and VIM (Additional files 1: Table S3, Table S4), or using the CV of fasting plasma glucose, instead of HbA1c CV (Additional file 1: Table S5)**.**

## Discussion

In this secondary analysis of data from ACCORD, we first observed that there were graded trends in the risk of mortality with the increasing numbers of higher CVs of the three variables: LDL-C, HbA1c, and HDL-C. Those with higher CVs of all three variables had the highest risks of cardiovascular and all-cause mortality.

Our current study examined the interactions of VVVs in LDL-C, HDL-C, and HbA1c on mortality and found that the VVVs of HbA1c and HDL-C were important effect modifiers of the LDL-C CV & mortality association. Firstly, a stronger positive association between LDL-C CV and mortality was found in those participants with higher HbA1c CV. Greater glycemic variability was associated with upregulation of stress hormones, activation of inflammation cascade, downstream oxidative stress and endothelial dysfunction [18–20]. Moreover, high HbA1c variability was linked to an increased risk of severe hypoglycemic episodes [21], which may underlie the increased mortality risk [22]. Indeed, HbA1c variability had been reported to be associated with a higher risk of CVD and mortality [8, 9, 19]. Secondly, the positive association between LDL-C variability and risk of CVD mortality was more pronounced in participants with higher HDL-C CV. Previous studies also have linked HDL-C CV with adverse clinic outcomes [23, 24]. Furthermore, those with a higher CV of all the three variables, including LDL-C, HbA1c, and HDL-C had the significant and highest risks of all-cause and CVD mortality, suggesting that the three CVs may enhance each other to increase the risk of mortality. This is not the first study to suggest joint effects of multiple risk factors variability. For example, Kwon et al. [25] reported that high variabilities of SBP, BMI, fasting blood glucose, and total cholesterol level was synergistically associated with a higher incidence of new-onset heart failure. However, our results were just hypothesis generation, and thus more studies are needed to verify our results and to further examine the biological mechanisms underlying the associations.

Our study had some limitations. First, our study is a post hoc analysis of the ACCORD trial. Despite the adjustments for a broad set of covariates, we cannot exclude the possibility of residual or unmeasured confounding. Second, the current study was limited in the lipid arm due to the limited measurements of lipids in the non-lipid arm. Third, the study population was derived from the ACCORD lipid trial, which included patients with type 2 diabetes who had prevalent CVD or risk factors and were undertaken lipid control. Thus, the findings may not be generalizable to patients who are at lower risk for CVD outside a clinical trial setting. Therefore, confirmation of our findings in future studies is essential.

## Conclusion

In conclusion, VVVs in LDL-C, HDL-C, and HbA1c may jointly affect the risks of cardiovascular and all-cause mortality in diabetes patients. Those with a higher CV of all three variables had the highest risks of cardiovascular and all-cause mortality. If further confirmed, our findings highlight the importance of substantial fluctuations in LDL-C, HDL-C, and HbA1c among patients with type 2 diabetes in forecasting future risk of mortality. Moreover, whether reducing the fluctuations in these parameters may lower mortality risk among type 2 diabetes patients should be further examined in future clinical trials.

## Supplementary Information

**Additional file 1: Figure S1.** Flow chart of study participants. **Table S1.** Associations between LDL-C CV and the study outcomes. **Figure S2.** Relations between LDL-C ARV, LDL-C VIM and the study outcomes. **Table S2.** Stratified analyses by potential effect modifiers for the association between LDL-C CV and CVD mortality in various subgroups. **Figure S3.** Stratified analyses by potential effect modifiers for the association between LDL-C CV and CVD mortality in various subgroups. **Figure S4.** Stratified analyses by potential effect modifiers for the association between LDL-C CV and all-cause mortality in various subgroups. **Table S3.** Associations between numbers of higher VIM of the three variables (LDL-C, HbA1c, and HDL-C) and the study outcomes. **Table S4.** Associations between numbers of higher ARV of the three variables (LDL-C, HbA1c, and HDL-C) and the study outcomes. **Table S5.** Associations between numbers of higher CV of the three variables (LDL-C, fasting glucose, and HDL-C) and the study outcomes.

## Data Availability

The data are available from the Biologic Specimen and Data Repository Information Coordinating Center (BioLINCC), National Heart, Lung and Blood Institute, U.S. Department of Health & Human Services. The analytic code will be made available from the corresponding authors on request.

## Abbreviations

VVV: Visit-to-visit variability
LDL-C: Low-density lipoprotein cholesterol
HDL-C: High-density lipoprotein cholesterol
CV: Coefficient of variation
ARV: Average real variability
VIM: Variability independent of the mean
CVD: Cardiovascular disease
ACCORD: Action to Control Cardiovascular Risk in Diabetes
SD: Standard deviation
BMI: Body mass index
SBP: Systolic blood pressure
DBP: Diastolic blood pressure
eGFR: Estimated glomerular filtration rate
