# Supplementary material for: Joint effect of visit-to-visit variability in LDL-cholesterol, HDL-cholesterol and HbA1c on cardiovascular and total mortality in patients with diabetes
Source: Diabetol Metab Syndr. 2022 Sep 19;14:132. doi: 10.1186/s13098-022-00905-x (PMC9487118; doi:10.1186/s13098-022-00905-x)
Supplement: Supplementary file 1 — Additional file 1: Figure S1. Flow chart of study participants. Table S1. Associations between LDL-C CV and the study outcomes. Figure S2. Relations between LDL-C ARV, LDL-C VIM and the study outcomes. Table S2. Stratified analyses by potential effect modifiers for the association between LDL-C CV and CVD mortality in various subgroups. Figure S3. Stratified analyses by potential effect modifiers for the association between LDL-C CV and CVD mortality in various subgroups. Figure S4. Stratified analyses by potential effect modifiers for the association between LDL-C CV and all-cause mortality in various subgroups. Table S3. Associations between numbers of higher VIM of the three variables (LDL-C, HbA1c, and HDL-C) and the study outcomes. Table S4. Associations between numbers of higher ARV of the three variables (LDL-C, HbA1c, and HDL-C) and the study outcomes. Table S5. Associations between numbers of higher CV of the three variables (LDL-C, fasting glucose, and HDL-C) and the study outcomes. [file 13098_2022_905_MOESM1_ESM.docx]

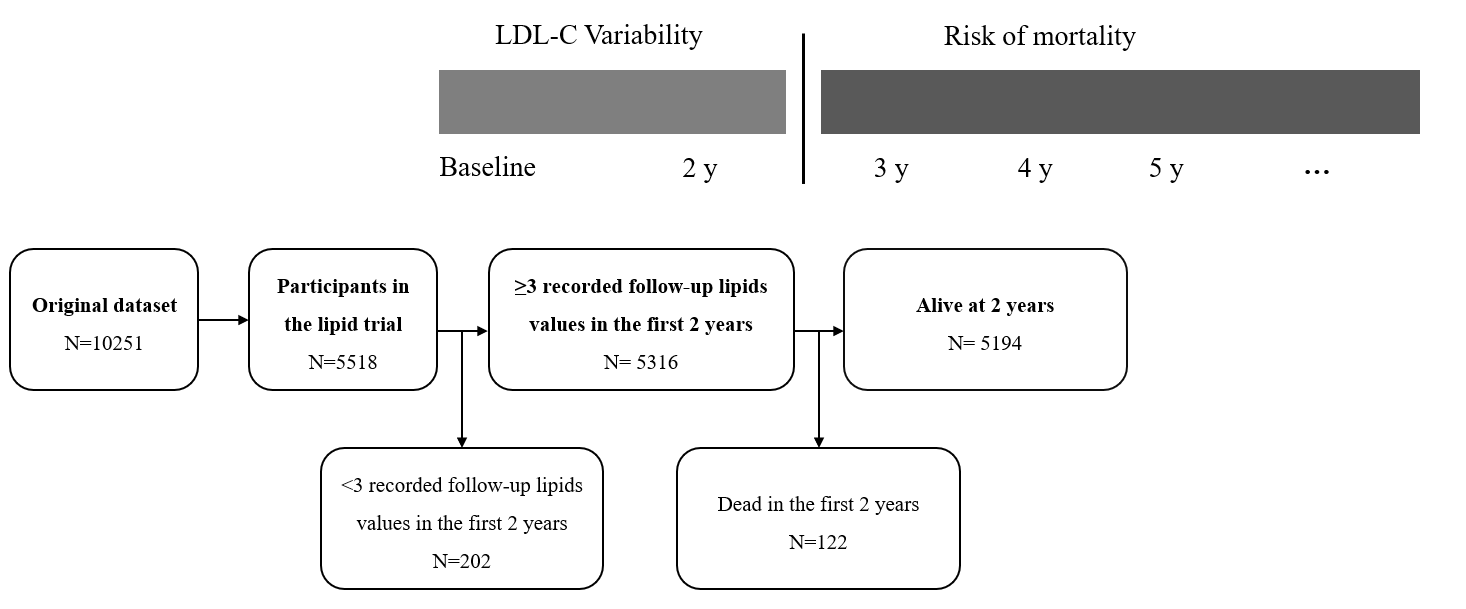


**Supplemental Figure 1. Flow chart of study participants**

**Supplemental Table 1. Associations between LDL-C CV and the study outcomes**

| **LDL-C CV, %** | **N** | **Event (%)** | **Crude Models** | | **Adjusted Models*** | |
| --- | --- | --- | --- | --- | --- | --- |
|  |  |  | **HR (95%CI)** | **P value** | **HR (95%CI)** | **P value** |
| **CVD mortality** |  |  |  |  |  |  |
| Continuous (Per SD increase) | 5194 | 144(2.8) | 1.27(1.09,1.47) | 0.002 | 1.32(1.12,1.55) | 0.001 |
| Quartiles |  |  |  |  |  |  |
| Q1 (<12.5) | 1299 | 33(2.5) | Ref |  | Ref |  |
| Q2 (12.5-<18.3) | 1298 | 32(2.5) | 0.97(0.59,1.57) | 0.891 | 1.02(0.62,1.67) | 0.944 |
| Q3 (18.3-<26.0) | 1298 | 30(2.3) | 0.90(0.55,1.48) | 0.681 | 0.91(0.55,1.52) | 0.723 |
| Q4 (≥26.0) | 1299 | 49(3.8) | 1.59(1.02,2.47) | 0.040 | 1.74(1.10,2.76) | 0.019 |
| Categories |  |  |  |  |  |  |
| Q1-3(<26.0) | 3895 | 95(2.4) | Ref |  | Ref |  |
| Q4 (≥26.0) | 1299 | 49(3.8) | 1.66(1.18,2.35) | 0.004 | 1.78(1.24,2.56) | 0.002 |
| **All-cause mortality** | |  |  |  |  |  |
| Continuous (Per SD increase) | 5194 | 305(5.9) | 1.19(1.07,1.32) | 0.001 | 1.22(1.09,1.36) | 0.001 |
| Quartiles |  |  |  |  |  |  |
| Q1 (<12.5) | 1299 | 71(5.5) | Ref |  | Ref |  |
| Q2 (12.5-<18.3) | 1298 | 64(4.9) | 0.90(0.64,1.26) | 0.527 | 0.93(0.66,1.31) | 0.670 |
| Q3 (18.3-<26.0) | 1298 | 70(5.4) | 0.97(0.70,1.35) | 0.865 | 0.99(0.71,1.38) | 0.938 |
| Q4 (≥26.0) | 1299 | 100(7.7) | 1.50(1.11,2.04) | 0.009 | 1.57(1.14,2.15) | 0.005 |
| Categories |  |  |  |  |  |  |
| Q1-3(<26.0) | 3895 | 205(5.3) | Ref |  | Ref |  |
| Q4 (≥26.0) | 1299 | 100(7.7) | 1.57(1.24,2.00) | < 0.001 | 1.61(1.26,2.07) | < 0.001 |

*Adjusted for sex, glycemia treatment group, lipids treatment group, race, age, education, BMI, diabetes duration, systolic blood pressure (SBP), smoking and drinking status, estimated glomerular filtration rate (eGFR) at baseline, as well as mean of HbA1c, high-density lipoprotein cholesterol (HDL-C), triglyceride (TG), low-density lipoprotein cholesterol (LDL-C) during the first 2 years of follow-up.


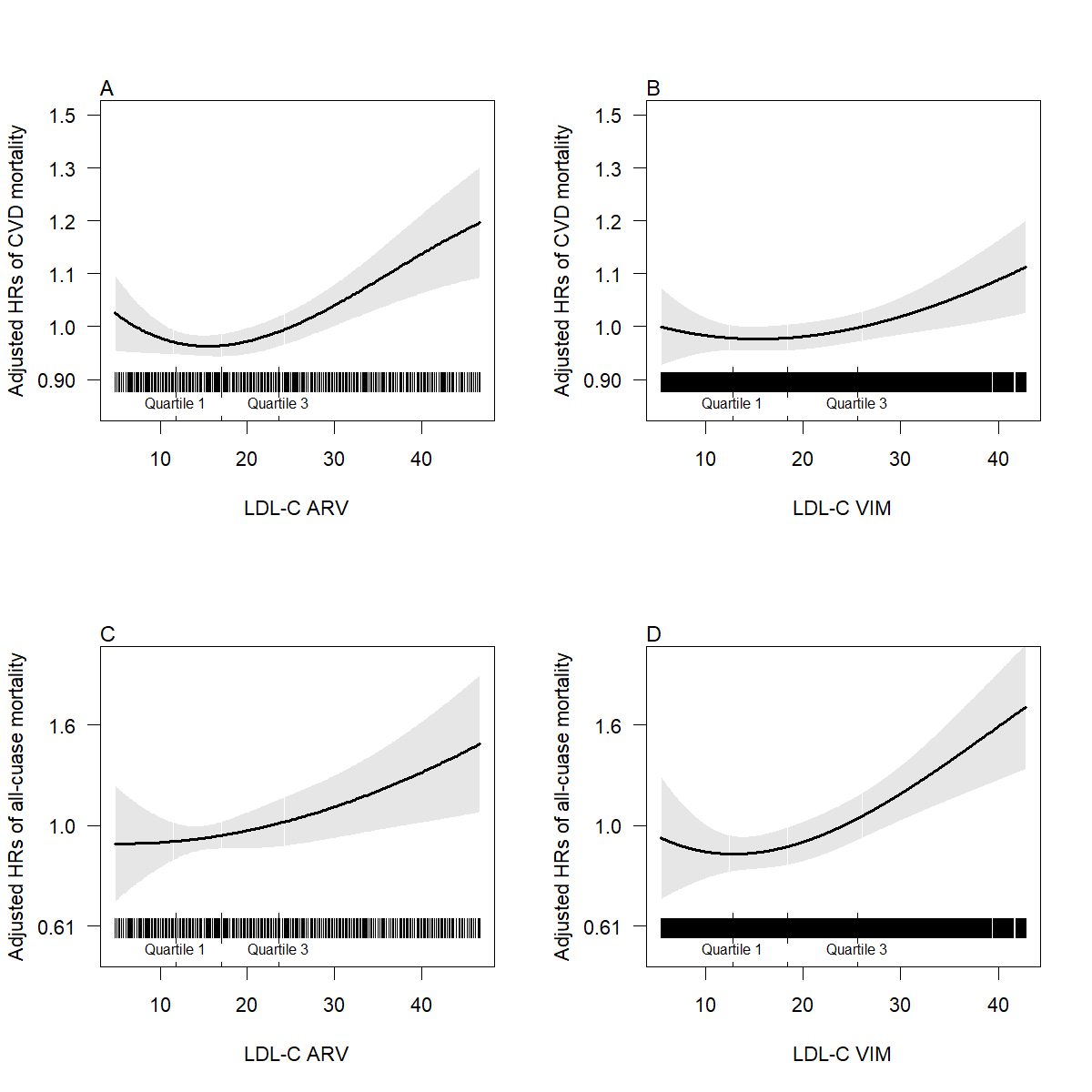


**Supplemental Figure 2. Relations between LDL-C ARV, LDL-C VIM and the study outcomes***

* Adjusted for sex, glycemia treatment group, lipids treatment group, race, age, education, BMI, diabetes duration, systolic blood pressure (SBP), smoking and drinking status, estimated glomerular filtration rate (eGFR) at baseline, as well as mean of HbA1c, high-density lipoprotein cholesterol (HDL-C), triglyceride (TG), low-density lipoprotein cholesterol (LDL-C) during the first 2 years of follow-up.

**Supplemental Table 2. Stratified analyses by potential effect modifiers for the association between LDL-C CV and CVD mortality in various subgroups***

| Subgroup | Crude Models | | Adjusted Models | |
| --- | --- | --- | --- | --- |
|  | HR (95%CI) | P for interaction | HR (95%CI) | P for interaction |
| At baseline |  |  |  |  |
| Sex |  | 0.707 |  | 0.753 |
| Male | 1.67(1.13,2.46) |  | 1.67(1.13,2.46) |  |
| Female | 1.96(0.93,4.14) |  | 1.91(0.90,4.04) |  |
| Age, years |  | 0.891 |  | 0.854 |
| <62 | 1.62(0.89,2.96) |  | 1.66(0.91,3.04) |  |
| ≥62 | 1.70(1.12,2.60) |  | 1.78(1.17,2.71) |  |
| BMI, kg/m^2 |  | 0.893 |  | 0.924 |
| <30 | 1.72(1.00,2.95) |  | 1.76(1.02,3.01) |  |
| ≥30 | 1.64(1.05,2.57) |  | 1.70(1.08,2.66) |  |
| During the first 2 years | |  |  |  |
| HbA1c CV, % |  | 0.014 |  | 0.014 |
| <5.7 (median) | 0.95(0.52,1.77) |  | 0.99(0.53,1.83) |  |
| ≥5.7 | 2.39(1.55,3.70) |  | 2.47(1.60,3.82) |  |
| HDL-CV, % |  | 0.026 |  | 0.026 |
| <12.8 (Q1-3) | 1.31(0.85,2.02) |  | 1.31(0.85,2.02) |  |
| ≥12.8 | 3.11(1.65,5.85) |  | 3.11(1.65,5.85) |  |
| TG CV, % |  | 0.387 |  | 0.334 |
| <18.7 (median) | 1.46(0.88,2.43) |  | 1.47(0.89,2.45) |  |
| ≥18.7 | 1.99(1.23,3.24) |  | 2.08(1.28,3.39) |  |
| HbA1c MEAN, % |  | 0.997 |  | 0.974 |
| <7.1 (median) | 1.70(1.03,2.82) |  | 1.77(1.07,2.93) |  |
| ≥7.1 | 1.70(1.05,2.73) |  | 1.75(1.08,2.81) |  |
| LDL-C MEAN, mg/dL | | 0.949 |  | 0.876 |
| <90.4 (median) | 1.66(0.99,2.81) |  | 1.70(1.01,2.87) |  |
| ≥90.4 | 1.70(1.07,2.70) |  | 1.80(1.13,2.86) |  |
| HDL-C MEAN, mg/dL | | 0.227 |  | 0.243 |
| <39.2 (median) | 1.37(0.86,2.20) |  | 1.42(0.89,2.28) |  |
| ≥39.2 | 2.11(1.26,3.53) |  | 2.16(1.29,3.61) |  |
| TG MEAN, mg/dL |  | 0.864 |  | 0.717 |
| <147.4 (median) | 1.72(1.08,2.73) |  | 1.83(1.15,2.91) |  |
| ≥147.4 | 1.62(0.96,2.71) |  | 1.61(0.96,2.70) |  |

*Adjusted, if not stratified, for sex, age.

**
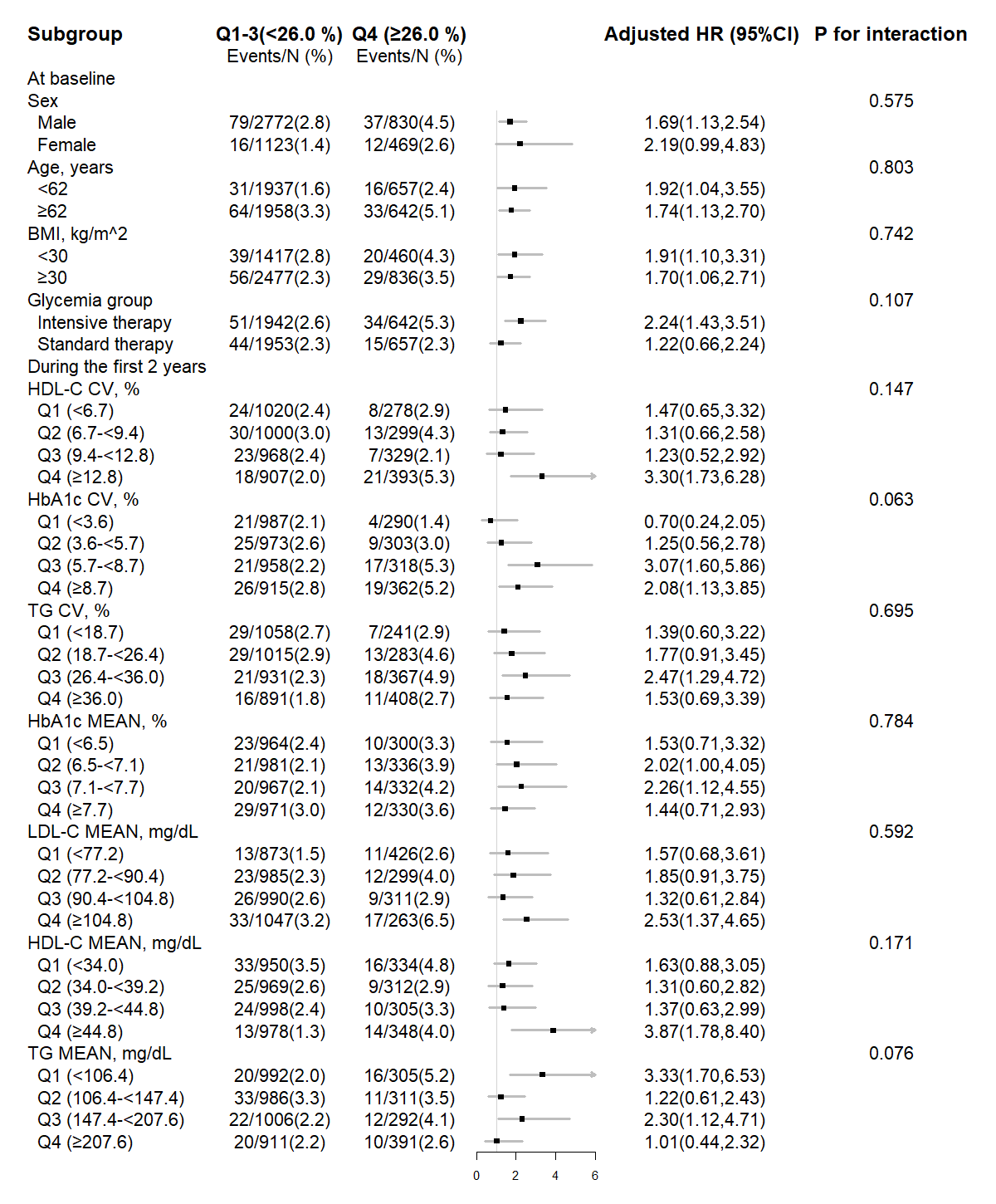
**

**Supplemental Figure 3. Stratified analyses by potential effect modifiers for the association between LDL-C CV and CVD mortality in various subgroups***

*Adjusted, if not stratified, for sex, glycemia treatment group, lipids treatment group, race, age, education, BMI, diabetes duration, systolic blood pressure (SBP), smoking and drinking status, estimated glomerular filtration rate (eGFR) at baseline, as well as mean of HbA1c, high-density lipoprotein cholesterol (HDL-C), triglyceride (TG), low-density lipoprotein cholesterol (LDL-C) during the first 2 years of follow-up.


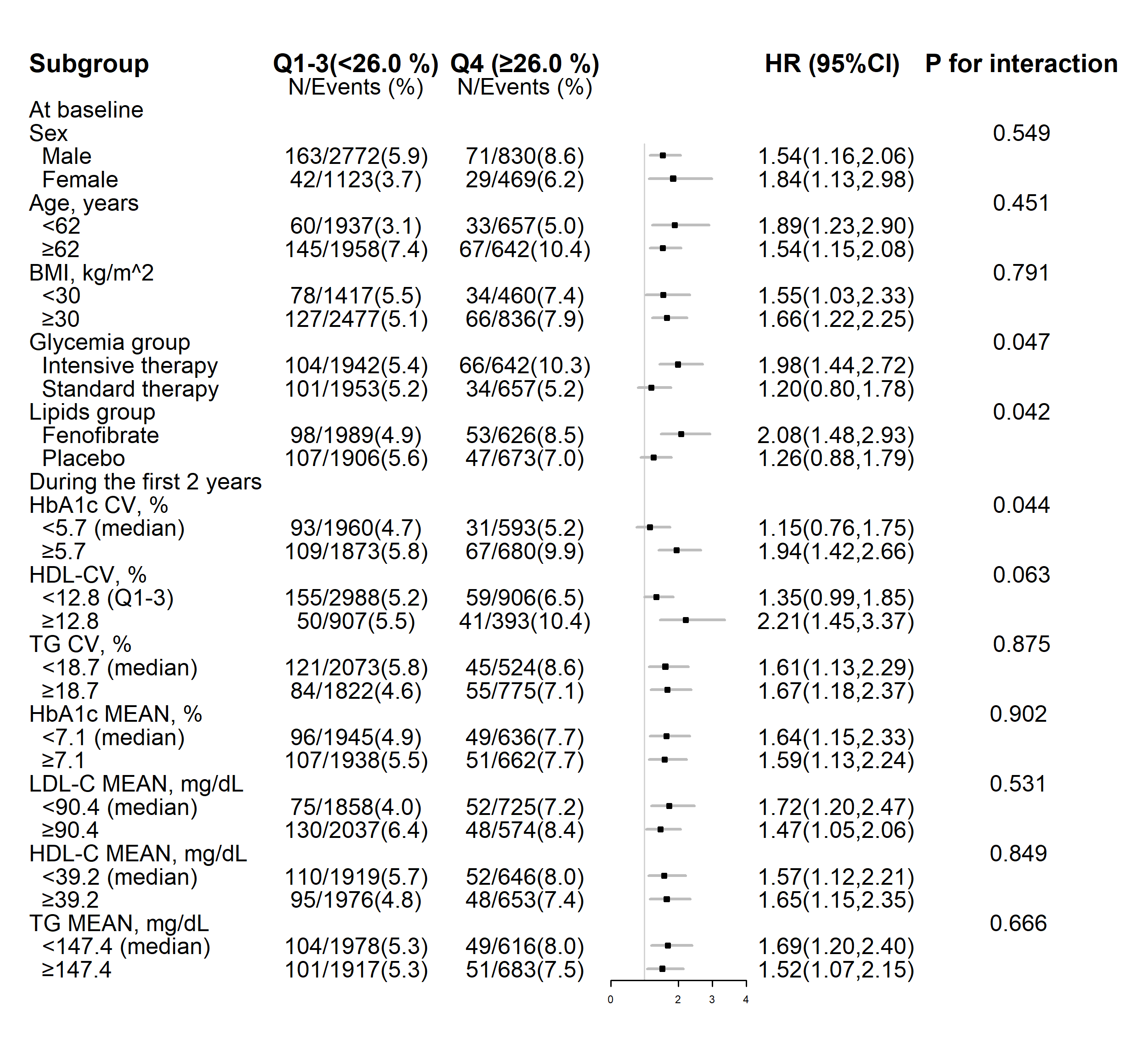


**Supplemental Figure 4.** **Stratified analyses by potential effect modifiers for the association between LDL-C CV and all-cause mortality in various subgroups***

*Adjusted, if not stratified, for sex, glycemia treatment group, lipids treatment group, race, age, education, BMI, diabetes duration, systolic blood pressure (SBP), smoking and drinking status, estimated glomerular filtration rate (eGFR) at baseline, as well as mean of HbA1c, high-density lipoprotein cholesterol (HDL-C), triglyceride (TG), low-density lipoprotein cholesterol (LDL-C) during the first 2 years of follow-up.

**Supplemental Table 3. Associations between numbers of higher VIM of the three variables (LDL-C, HbA1c, and HDL-C) and the study outcomes***

| **Number of higher VIM** | **N** | **Event (%)** | **Crude Models** | | **Adjusted Models** | |
| --- | --- | --- | --- | --- | --- | --- |
|  |  |  | **HR (95%CI)** | **P value** | **HR (95%CI)** | **P value** |
| **CVD mortality** | |  |  |  |  |  |
| 0 | 1581 | 38(2.4) | Ref |  | Ref |  |
| 1 | 2182 | 56(2.6) | 1.06(0.71,1.61) | 0.765 | 1.06(0.70,1.61) | 0.794 |
| 2 | 1112 | 31(2.8) | 1.16(0.72,1.87) | 0.537 | 1.17(0.72,1.91) | 0.524 |
| 3 | 231 | 17(7.4) | 3.04(1.72,5.39) | < 0.001 | 3.08(1.68,5.62) | < 0.001 |
| P for trend | |  | 0.007 |  | 0.010 |  |
| **All-cause mortality** | |  |  |  |  |  |
| 0 | 1581 | 71(4.5) | Ref |  | Ref |  |
| 1 | 2182 | 127(5.8) | 1.29(0.97,1.73) | 0.085 | 1.28(0.95,1.71) | 0.105 |
| 2 | 1112 | 73(6.6) | 1.46(1.05,2.02) | 0.024 | 1.48(1.06,2.07) | 0.021 |
| 3 | 231 | 29(12.6) | 2.80(1.82,4.31) | < 0.001 | 2.75(1.76,4.30) | < 0.001 |
| P for trend | |  | < 0.001 |  | < 0.001 |  |

*****Adjusted for sex, glycemia treatment group, lipids treatment group, race, age, education, BMI, diabetes duration, systolic blood pressure (SBP), smoking and drinking status, estimated glomerular filtration rate (eGFR) at baseline, as well as mean of HbA1c, high-density lipoprotein cholesterol (HDL-C), triglyceride (TG), low-density lipoprotein cholesterol (LDL-C) during the first 2 years of follow-up.

**Supplemental Table 4. Associations between numbers of higher ARV of the three variables (LDL-C, HbA1c, and HDL-C) and the study outcomes***

| **Number of higher ARV** | **N** | **Event (%)** | **Crude Models** | | **Adjusted Models** | |
| --- | --- | --- | --- | --- | --- | --- |
|  |  |  | **HR (95%CI)** | **P value** | **HR (95%CI)** | **P value** |
| **CVD mortality** | |  |  |  |  |  |
| 0 | 1464 | 38(2.6) | Ref |  | Ref |  |
| 1 | 2287 | 57(2.5) | 0.94(0.62,1.41) | 0.749 | 0.92(0.60,1.40) | 0.685 |
| 2 | 1111 | 37(3.3) | 1.24(0.79,1.95) | 0.351 | 1.19(0.73,1.93) | 0.478 |
| 3 | 244 | 10(4.1) | 1.48(0.74,2.98) | 0.268 | 1.58(0.76,3.29) | 0.219 |
| P for trend |  |  | 0.178 |  | 0.216 |  |
| **All-cause mortality** | |  |  |  |  |  |
| 0 | 1464 | 72(4.9) | Ref |  | Ref |  |
| 1 | 2287 | 129(5.6) | 1.11(0.83,1.49) | 0.463 | 1.11(0.83,1.50) | 0.473 |
| 2 | 1111 | 81(7.3) | 1.42(1.04,1.95) | 0.030 | 1.45(1.04,2.03) | 0.029 |
| 3 | 244 | 18(7.4) | 1.40(0.84,2.35) | 0.199 | 1.56(0.91,2.66) | 0.107 |
| P for trend |  |  | 0.024 |  | 0.016 |  |

*****Adjusted for sex, glycemia treatment group, lipids treatment group, race, age, education, BMI, diabetes duration, systolic blood pressure (SBP), smoking and drinking status, estimated glomerular filtration rate (eGFR) at baseline, as well as mean of HbA1c, high-density lipoprotein cholesterol (HDL-C), triglyceride (TG), low-density lipoprotein cholesterol (LDL-C) during the first 2 years of follow-up.

**Supplemental Table 5. Associations between numbers of higher CV of the three variables (LDL-C, fasting glucose, and HDL-C) and the study outcomes***

| **Number of higher CV** | **N** | **Event (%)** | **Crude Models** | | **Adjusted Models** | |
| --- | --- | --- | --- | --- | --- | --- |
|  |  |  | **HR (95%CI)** | **P value** | **HR (95%CI)** | **P value** |
| **CVD mortality** | |  |  |  |  |  |
| 0 | 1573 | 25(1.6) | Ref |  | Ref |  |
| 1 | 2278 | 71(3.1) | 1.95(1.24,3.08) | 0.004 | 1.69(1.06,2.70) | 0.028 |
| 2 | 1111 | 33(3.0) | 1.93(1.15,3.25) | 0.013 | 1.66(0.97,2.85) | 0.065 |
| 3 | 232 | 15(6.5) | 4.49(2.36,8.51) | < 0.001 | 4.89(2.51,9.52) | < 0.001 |
| P for trend | |  | < 0.001 |  | < 0.001 |  |
| **All-cause mortality** | |  |  |  |  |  |
| 0 | 1573 | 61(3.9) | Ref |  | Ref |  |
| 1 | 2278 | 142(6.2) | 1.60(1.18,2.16) | 0.002 | 1.48(1.09,2.02) | 0.012 |
| 2 | 1111 | 75(6.8) | 1.80(1.28,2.52) | 0.001 | 1.67(1.18,2.36) | 0.004 |
| 3 | 232 | 27(11.6) | 3.30(2.09,5.19) | < 0.001 | 3.44(2.15,5.50) | < 0.001 |
| P for trend | |  | < 0.001 |  | < 0.001 |  |

*****Adjusted for sex, glycemia treatment group, lipids treatment group, race, age, education, BMI, diabetes duration, systolic blood pressure (SBP), smoking and drinking status, estimated glomerular filtration rate (eGFR) at baseline, as well as mean of HbA1c, high-density lipoprotein cholesterol (HDL-C), triglyceride (TG), low-density lipoprotein cholesterol (LDL-C) during the first 2 years of follow-up.
